# Intravitreal injection of peptides PnPa11 and PnPa13, derivatives of *Phoneutria nigriventer* spider venom, prevents retinal damage

**DOI:** 10.1590/1678-9199-JVATITD-2020-0031

**Published:** 2020-09-23

**Authors:** Lays Fernanda Nunes Dourado, Flavia Rodrigues da Silva, Cibele Rodrigues Toledo, Carolina Nunes da Silva, Cleildo Pereira Santana, Bruna Lopes da Costa, Maria Elena de Lima, Armando da Silva Cunha

**Affiliations:** 1 School of Pharmacy, Federal University of Minas Gerais (UFMG), Belo Horizonte, MG, Brazil.; 2 National Institute of Science and Technology in Pharmaceutical Nanotechnology, São Paulo, SP, Brazil.; 3 Graduate Program in Health Sciences: Medicine and Biomedicine, Institute of Education and Research, Santa Casa de Belo Horizonte, Belo Horizonte, MG, Brazil.

**Keywords:** Phoneutria nigriventer, Neuroprotection, Retinal diseases, Toxicity, Blue LED, Synthetic peptides

## Abstract

**Background:**

PnPa11 and PnPa13 are synthetic peptides derived from *Phoneutria nigriventer* spider venom, which display antinociceptive and neuroprotective properties. In this work, we evaluated the safety of intravitreal use and the neuroprotective effect of these peptides.

**Methods:**

The cytotoxicity and the antiangiogenic activity of these peptides were evaluated by the sulforhodamine-B method and chicken chorioallantoic membrane (CAM) assay, respectively. The *in vivo* safety was analyzed in Wistar rats that were intravitreally injected with different doses (0.50; 1.25; 2.50; 3.75 and 5.00 µg/mL) of these peptides (right eye, n = 6). The retinal function was assessed by electroretinography exams (ERG), intraocular pressure (IOP), and histological analyzes. In order to investigate the neuroprotective effect, Wistar rats received intravitreal injections (right eye, n = 6) of peptides at 1.25 µg/mL and then were exposed to blue LED light. In addition, the visual function and the retinal microstructure were verified.

**Results:**

Cytotoxicity analyses demonstrated that the peptides did not present any toxicity over ARPE-19 (adult retinal pigmented epithelial) cell line and the antiangiogenic study highlighted that the peptides promoted the reduction of blood vessels. The intravitreal injection did not cause major changes, neither induced any irreversible damage. In the retinal degeneration assay, the ERG records demonstrated that the prior treatment with PnPa11 and PnPa13 protected the retina from damage. Morphological analyses confirmed the ERG findings. Immunoblotting analyses revealed that PnPa11 increased Erk1/2, NR2A, and NR2B retinal expression after the light stress model, but did not cause Akt1 activation, while PnPa13 prevented Erk1/2 and Akt1 dephosphorylation.

**Conclusions:**

The intraocular administration of these peptides was well tolerated and presented protective activity against retinal degeneration, suggesting the potential use of these peptides as neuroprotectors in the ophthalmological field.

## Background

Photoreceptor degeneration and apoptosis are important pathological processes in retinal neurodegeneration [1,2,3]. These alterations can cause blindness and consequently have a detrimental impact on quality of life [4]. Population-based investigations have pointed out the high prevalence of neurodegenerative diseases of the eye such as age-related macular degeneration (AMD), retinitis pigmentosa, and glaucoma [5,6]. Moreover, with the aging of the population, the number of people who develop some kind of neurodegenerative disease is rapidly growing [7]. 

Previous studies have shown that acute exposure to light can promote apoptosis of retinal pigmented epithelium cells and photoreceptors, intensifying the progression of neurodegenerative diseases of the eye [8], and several studies have demonstrated that blue, or blue-rich white LEDs, are more toxic because they can initiate damage and death of photoreceptors more easily [9,10,11,12].

Research for candidate molecules extracted from venomous animals has been intense during the last decades and contributes to the development of new drugs. Spider venoms, including that of *Phoneutria nigriventer*, are rich in protein and peptide toxins that have an affinity for a wide range of tissue receptors [13,14,15]. A valuable tool to investigate the neurodegeneration process is the alteration of N-methyl-D-aspartate (NMDA) receptors, once glutamate is the main excitatory neurotransmitter in the retina. Interestingly, some toxins from *P. nigriventer’s* venom, such as PnTx4(6-1) and PnTx4(5-5), can inhibit the glutamate uptake [16]. 

PnTx4 (6-1), δ-ctenitoxin-Pn1a, is a peptide composed of 48 amino acid residues, with a molecular mass of 5.2 kDa. According to the study conducted by Mafra and collaborators, PnTx4 (6-1) can inhibit the glutamate uptake in rat cerebro-cortical synaptosomes [17]. PnTx4(5-5), also called Γ-ctenitoxin-Pn1a, consists of 47 amino acid residues, including 10 cysteines, with a molecular mass of 5.175 kDa, and it acts as a reversible antagonist of NMDA ionotropic glutamate receptor in rat brain neurons [18,19].

One of the biggest problems in designing new animal studies using spider toxins is the limitation of the amount of material when compared to the quantities needed for pharmacological assays. Moreover, natural toxins frequently are complex molecules, with limited tissue absorption, and due to the several disulfide bridges, their syntheses are very difficult [20]. However, thanks to the rational study of these toxins, the identification of active regions is done by using databases and molecular modeling tools. These studies are useful to obtain new and smaller peptides, that are easier to be chemically synthesized and tested in different assays [21].

In this work, the two synthetic peptides employed have been obtained by Immune Epitope Database and Analysis Resource program: PnPa11 (*P. nigriventer* peptide antinociceptive, containing 11 amino acid residues, SEQ ID N°1 -DCYWSDSCKSR) and PnPa13 (*P. nigriventer* peptide antinociceptive, containing 13 amino acid residues, SEQ ID N°1- H-CDSYWSKSSKCRE-NH2). Their sequences were based on studies of the toxins PnTx4(5-5) and PnTx4(6-1), respectively [22,23]. These peptides are potential molecules to promote the development of new pharmaceutical compositions [16,21].

Considering the lack of studies evaluating the toxicity and safety of ocular application of these synthetic peptides, the present work aimed to assess the viability of using these synthetic peptides in the eye, and also to explore the neuroprotector effect of PnPa11 and PnPa13.

## Methods

### Peptides

The synthetic peptides derived from *Phoneutria nigriventer* PnTx4(5-5) and PnTx4(6-1) were designed based on *in silico* studies. For the PnTx4(5-5) peptide, molecular docking was performed through the protein-protein anchor program, ClusPro. The PnTx4(5-5) residues supposedly interacting with the insect sodium channel (also predicted by modeling) were identified and subsequently submitted to a new docking, being the peptide with the highest interaction selected and synthesized. This peptide had 11 amino acid residues, exhibited cyclic conformation, and was named PnPa11. For the PnTx4(6-1) peptide, the epitopes prediction tool was used to detect the most exposed residues in the amino acid sequence. PnTx4 (6-1) had 13 amino acid residues a linear conformation, and it was named PnPa13. Both peptides were synthesized using solid-phase Fmoc strategy [24].

### Cytotoxicity evaluation

The ARPE-19, adult retinal pigmented epithelial cell line, (Cellular Bank of Rio de Janeiro, Brazil) was incubated following the methodology described by Toledo et al., 2019 [25]. For cell viability, sulforhodamine B (SRB) colorimetric assay was carried out [26]. About 10,000 cells/well were applied in 96-well plates. After 24 hours, the cells received the peptides PnPa11 and PnPa13 at the following concentrations: 0.25; 0.5; 1.25; 2.5; 3.75; 5.0; 8.0, 12.5 and 25.0 µg/mL, and the plate was incubated for 48hours. The medium was replaced, and the cells were fixed with 10% (v/v) trichloroacetic acid (Sigma Aldrich, USA). Subsequently, the cells were rinsed with water and stained with 0.057% (v/v) SRB solution (Sigma Aldrich, USA) in 1% (v/v) acetic acid (HAc) for 30 minutes at 30°C. Thereafter, the cells were rinsed with 1% (v/v) HAc, then incubated with tris base, 110 mM, pH 10.5 (Sigma Aldrich, USA), and shaken for 5 min. Absorbance was measured (510 nm), using a microplate reader (Bio-rad, San Diego, CA). Three wells per dose were used in three independent experiments. The cell viability was calculated as a percentage of the control. Besides, morphological changes in the cells were observed (5x) using a microscope (Axio Imager M2; ZEISS, Germany). 

### Chick embryo chorioallantoic membrane (CAM) assay

Fertilized hen eggs (*Gallus gallus domesticus*) were incubated at 37 ºC and 60% of relative air humidity (Premium Ecológica, Brazil). On the 3^rd^ day, a small hole (1 cm²) was made in the eggshell and the inner shell membrane was removed to expose the CAM. The hole was closed with transparent tape and then, the eggs returned to the incubator for 48 hours more. The eggs were uncovered and PnPa11 and PnPa13 peptide suspensions of 0.50, 1.25, 2.50, 3.75 e 5.00 µg/mL were applied (50 μL) on the 5^th^ and 6^th^ day, respectively. A saline solution (0.9% w/v NaCl) was set as the negative control and bevacizumab (250 µg/mL) was set as the positive control. Ten eggs per group were used. On the 7^th^ day, the membranes were photographed (model DM4000B, Leica, Germany - digital CCD camera model DFC 280).

To convert the images to grayscale, the microphotographs were processed using the ImageJ^TM^ software (version 1.50i - National Institutes of Health, USA). Subsequently, a quantitative analysis of the vascular network was carried out using the Angiotool^TM^ software (National Cancer Institute, USA). With the aid of this software, we can investigate the differences regarding vessel percentage area, lacunarity, and total number of junctions. The saline group was set to 100%.

### Animals

Adult male Wistar rats, aged 7 weeks and weighing 200 g, were kept with controlled conditions of temperature (27 ± 5 ºC) and luminosity (12 hours light/12 hours dark). The animals remained without restriction to water or food.

The *in vivo* studies were approved by the Ethics Committee in Experimental Animals (Protocols nº 107/2018 and 325/2017). All tests were accomplished following the National Institutes of Health (NIH) guidelines for the care and use of Laboratory Animals [27] and the guidelines of the Association for Research in Vision and Ophthalmology (ARVO). 

### ***In vivo* toxicity study**

In order to investigate the intravitreal toxicity of the PaPn11 and PaPn13 peptides, the animals were anesthetized via intraperitoneal injection of 90mg/kg ketamine (Dopalen; Ceva, Brazil) plus 10 mg/kg xylazine hydrochloride (Anasedan; Ceva, Brazil). Sequentially, the right eyes were anesthetized using one drop of 0.5% (w/v) proxymetacaine hydrochloride (Anestalcon; Alcon, Brazil). For intravitreal injections, the animals were divided into three main groups: PnPa11 (n = 20), PnPa13 (n = 20) and vehicle (saline) (n = 4). The groups that received the synthetic peptide were subdivided according to the intravitreal concentration of the peptides administrated (0.50; 1.25; 2.50; 3.75 and 5.00 µg/mL) (n = 4). The left eyes of all animals were kept intact.

A 30-gauge needle attached to a syringe was inserted ∼2 mm to the limbus. Besides that, the needle was held in place for 30seconds to prevent it from escaping the application site. The volume of intravitreal injection was set to 10 µL [28]. Each concentration was calculated based on the dilution that occurs in the vitreous humor (for adult rats, the vitreous volume is about 50 μL) [29].

### ERG recordings

ERG examinations were performed before and 7 days after the intravitreal injection. After 12 hours of total dark adaptation, the animals were anesthetized as previously described (“*In vivo* toxicity study” section) and the pupils were dilated using one drop of 0.5% (w/v) tropicamide (Mydriacyl; Alcon, Brazil). Immediately before ERG records, the eyes were topically anesthetized with one drop of 0.5% (w/v) proxymetacaine hydrochloride (Anestalcon; Alcon, Brazil).

The ERGs were conducted using a computerized system (EspionE^2^ electrophysiology system) and an LED stimulator (Ganzfeld ColorDome™, Diagnosys LLC, USA). To obtain the visual responses, a bipolar contact lens electrode (ERG Jet; Fabrinal SA, Switzerland) was put above each cornea, two subcutaneous steel needle electrodes were put in the front, and a ground electrode on the back of each cornea. Impedance value was set to less than 5kΩ in each electrode. During the test, flashes of white light with a duration of 4 milliseconds (ms) were produced in 11 steps (0.003 - 3cd·s·m^−2^) of increasing luminosity. ERGs results were amplified and analyzed using Espion E³ software (Diagnosys LLC, MA). All procedure was carried out in compliance with the International Society for Clinical Electrophysiology of Vision (ISCEV) guidelines.

The ERGs results obtained were amplitude, expressed in microvolts (μV) and implicit time (ms) of scotopic a and b-waves. The a-wave amplitude was measured from the average, pre-stimulus baseline, to the a-wave trough. The b-wave amplitude was measured from a-wave trough to b-wave peak. The a-wave and b-wave implicit times were measured from the time of the flash to the peak of the wave [30]. Previous studies have shown that the most common stimulus to investigate the rat retinal responses are at 0.01 cd·s·m^−2^, to evaluate rods response, and at 3 cd·s·m^−2^, to analyze the combined responses of cones and rods [31,32].

### Fundus ophthalmoscopy and IOP monitoring

Indirect fundus ophthalmoscopy (Welch Allyn, USA) was performed in both eyes immediately before the intravitreal injections and after the ERG examinations (the animals were anesthetized). The IOP was monitored using a veterinary tonometer (Tono-Pen Vet; Reichert, USA). For the measurements, four IOP readings were taken for each right eye (n = 4). 

### Histological analysis

All animals were euthanized and the eyes were prepared for hematoxylin and eosin staining [23]. For the longitudinal sections, the eyes were embedded in paraffin, sectioned into 5 µm-thick, and stained with hematoxylin and eosin (Sigma-Aldrich, Germany). Retina morphology and the presence of inflammatory cells were evaluated under light microscopy, model Axio Imager M2 (ZEISS, Germany) equipped with a 20x objective lens. The outer nuclear layer (ONL) thickness was measured at 250 μm distance of the optic nerve. A total of three measures was done for each histological slide (n = 3).

### Retinal degeneration study

For the retinal degeneration study, Wistar rats were assigned into four groups: PnPa11 (1.25 µg/mL, n = 8), PnPa13 (1.25 µg/mL, n = 8), blue-LED (saline, n = 8) and healthy (without any procedure). Intravitreal injection was done in the right eye for the PnPa11, PnPa13, and vehicle groups, as previously described (“*In vivo* toxicity study” section). The left eyes of all animals were kept intact.

After intravitreal injection, all rats were maintained in a dark room for 12 hours. Then, the animals were exposed to the blue LED of 2000 lux intensity for 72 hours (6 days with 12 hours exposure each, respecting the light/dark cycle without restriction of food or water). For the exposure, lamps were affixed to the top of individual wooden cages, with dimensions of 57 cm x 57 cm x 60 cm. After the light stress procedure, the animals were dark-adapted for 12 hours. Sequentially, they were anesthetized and submitted to the full-field ERG for the evaluation of the retinal function (“ERG recordings” section). For the blue-LED study, ERG was conducted both before the intravitreal injection, and 1, 7 and 15 days after the blue LED light exposure (n = 8 per group). After the last ERG, the animals were euthanized, and the eyes were enucleated. The eyes were sectioned in the sagittal plane (next to the optic nerve) and one-half of the eyes were prepared as described before (“Histological analysis” section) using a total of three samples for each group. 

### Transmission electron microscopy (TEM)

TEM of retinal tissues after light-exposure was performed as reported by Chen and collaborators [33]. Briefly, the eyes (n = 2) were collected and fixed using 2.5% (v/v) glutaraldehyde solution (Sigma Aldrich, USA) at 20ºC for 2 hours. Then, the eyes were fixed with osmium 1%(v/v) tetroxide (Sigma Aldrich, USA) for 2 hours, followed by dehydration steps in ethanol and immersion in epon 812 (Sigma Aldrich, USA). The slices of retina were obtained and were examined using a high-resolution TEM instrument (Tecnai G2-12 - FEI Spirit Biotwin) at 120 kV (located at the Center of Microscopy, UFMG, Belo Horizonte, Minas Gerais, Brazil).

### Immunohistochemistry Terminal Transferase dUTP Nick End Labeling (TUNEL) assay

After the eye section (“Retinal degeneration study” section), the eyes were fixed in 8% (v/v) paraformaldehyde (Sigma Aldrich, USA) in phosphate-buffered saline (PBS), pH 7.4 for 12 hours. For cryosection, the eyes were initially washed (three times) in PBS and then transferred to a 20% (w/v) sucrose solution for 4 - 5 hours. Thereafter, for the cryoprotection, the eyes were immersed in 40% (w/v) sucrose solution for 12 hours. Lastly, the eyes were rinsed in PBS and incorporated into the optimal cutting temperature compound (OCT) (Tissue-Tek, Japan) and instantly frozen in liquid nitrogen. The samples were cut at -25 °C and with 30 μm of diameter in the sagittal plane using a CM1850 cryostat (Leica, Germany). The sections were prepared on silanized slides (Knittel, Brazil) and stored at - 80°C.

For TUNEL procedures, the slides were washed with 0.1% Triton X-100 (Sigma Aldrich, USA) in 0.1% (v/v) sodium citrate (Sigma Aldrich, USA) in PBS. The slides were rinsed and incubated with 3% (v/v) hydrogen peroxide for 15 minutes (Sigma Aldrich, USA). Subsequently, the eyes were extensively flushed with PBS and submitted to the TUNEL assay (Roche, USA) following the manufacturer's protocol. Immunofluorescence was observed using a laser-scanning confocal microscope LSM 880 (ZEISS, Germany) located at the Center of Image Acquisition and Processing (CAPI/UFMG). Retinal sections were counterstained with DAPI (4´,6-Diamidin-2-phenylindol; Serva Electrophoresis, Germany). The immunofluorescence was observed using a laser-scanning confocal microscope LSM 880 (ZEISS, Germany). The photographs were taken with 40x lens at 250μm distance from the optic nerve. The intensity fluorescence of TUNEL-positive cells wasz calculated by ImageJ^TM^ software (n = 3 per group). For statistical analysis, the immunofluorescence intensity of the blue LED group was set to 100% and the immunofluorescence intensity of peptide-treated groups was compared to the blue-LED group. 

### Immunoblotting

Immunoblotting was performed according to Silva and collaborators [34]. A total of 100 µg of cellular protein (each sample, 3) was loaded in 12.5 and 4% (w/v) bys-acrylamide SDS-PAGE (Sigma Aldrich, USA) followed by electroblotting. Membranes were blocked with 0.5% of BSA, and sequentially incubated with primary antibodies overnight at 4ºC: rabbit anti-phospho-Akt1, 1:1000 (X20-A#127DB Bioteck™, Slovakia); total-Akt1, 1:1000 (C-20A#126/DB Bioteck™, Slovakia), phospho-Erk1/2, 1:1000 (MA515173, Invitrogen™, USA); total-Erk1/2 1:1000 (MA515134/Invitrogen™, USA); anti-NMDA 1:1000 (NR2B#pS1480/Invitrogen™, USA); anti-NMDA 1:1000 (NR2A#480031/ Invitrogen™, USA) and mouse anti-β-actin 1:1000 (Santa Cruz Laboratories, Brazil) antibodies in wash buffer 3% (w/v) BSA in PBS, pH 7.4. Membranes were washed 3 times during 5 minutes in PBS and maintained with either secondary horseradish peroxidase-conjugated goat anti-mouse (1:2500), or anti-rabbit IgG 1:3000 (BioRad Laboratories, USA). Membranes were washed 3 times during 10 minutes in PBS, and immersed with ECL Luminata (GE Healthcare, USA). Afterwards, non-saturated and immunoreactive bands were calculated by scanning densitometry using the Image Quant LAS software (GE Healthcare, USA). The immuno-band intensity was quantified by the ImageJ^TM^ software (version 1.49p National Institutes of Health, USA). The value of bands (number of pixels) was divided either by the value of β-actin or by the total-bands to normalize the expression or phosphorylation levels.

### Data analysis

For all analyses, mean and standard deviation (SD) values were determined. Means ± SD were shown for the number of independent experiments indicated in the figure captions. 

In cytotoxicity evaluation, CAM assay, histology and TUNEL analysis the data were performed using one-way ANOVA followed by posttest of Tukey, with p < 0.05 indicating significance.

To evaluate the normality of the pattern of ERG curves, Shapiro-Wilk test followed by Kruskal-Wallis and the post-test of Dunn was performed. To calculate the difference between amplitudes, implicit times of a and b-wave, intraocular pressure, Western blot analysis, two-way ANOVA followed by Bonferroni post-test with p < 0.05 indicating significance. Statistical analyses were performed using GraphPad Prism^TM^ v.5.0 software.

## Results

### PnPa11 and PnPa13 do not alter the viability of ARPE-19 cell line

**Figure 1.**
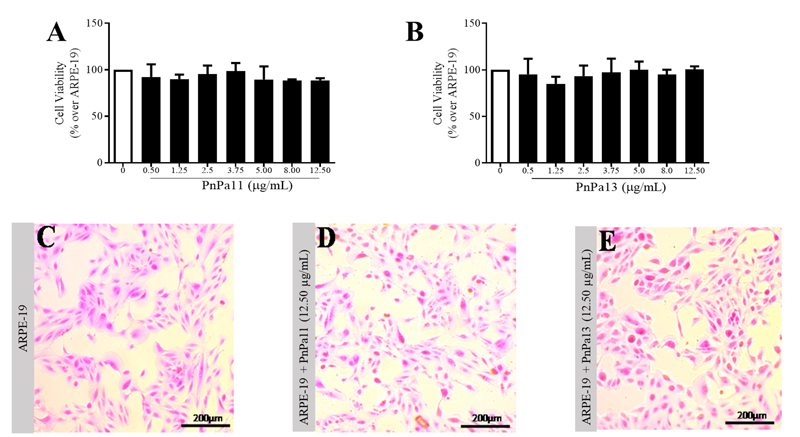
Cytotoxicity of PnPa11 and PnPa13 over ARPE-19 cells. **(A)** Visualization by optic microscopy of ARPE-19 cells after the treatment with PnPa11. **(B)** Visualization by optic microscopy of ARPE-19 cells after the treatment with PnPa13. **(C)** Visualization by optic microscopy of ARPE-19 cells. **(D)** Cell viability in the presence of increasing concentrations of PnPa11. **(E)** Cell viability in the presence of increasing concentrations of PnPa13. Comparison among groups was performed using one-way ANOVA with Tukey’s post-test (n = 3 well per dose in three independent experiments). The control group was set as 100%. The data are represented by mean ± SD. *Significant difference as compared to control (*p < 0.05).

According to our review, there is no previous *in vitro* study investigating the toxicity of venoms *P. nigriventer* as well as of its toxins or synthetic peptides derivatives on ARPE-19 cell culture. Therefore, we designed the *in vitro* study using low concentrations of PnPa11 and PnPa13 to evaluate the response of both synthetic peptides (0.50; 1.25; 2.50; 3.75; 5.00; 8.00 and 12.50 µg/mL). As shown in Figure 1A and 1B, no difference in cell viability was observed for groups treated with PnPa11 and PnPa13. Furthermore, in comparison to the control (Fig. 1C), no morphological alterations were observed in groups of cells treated with PnPa11 or PnPa13 (Fig. 1D and 1E). 

### PnPa11 and PnPa13 promote vascular reduction on CAM

**Figure 2.**
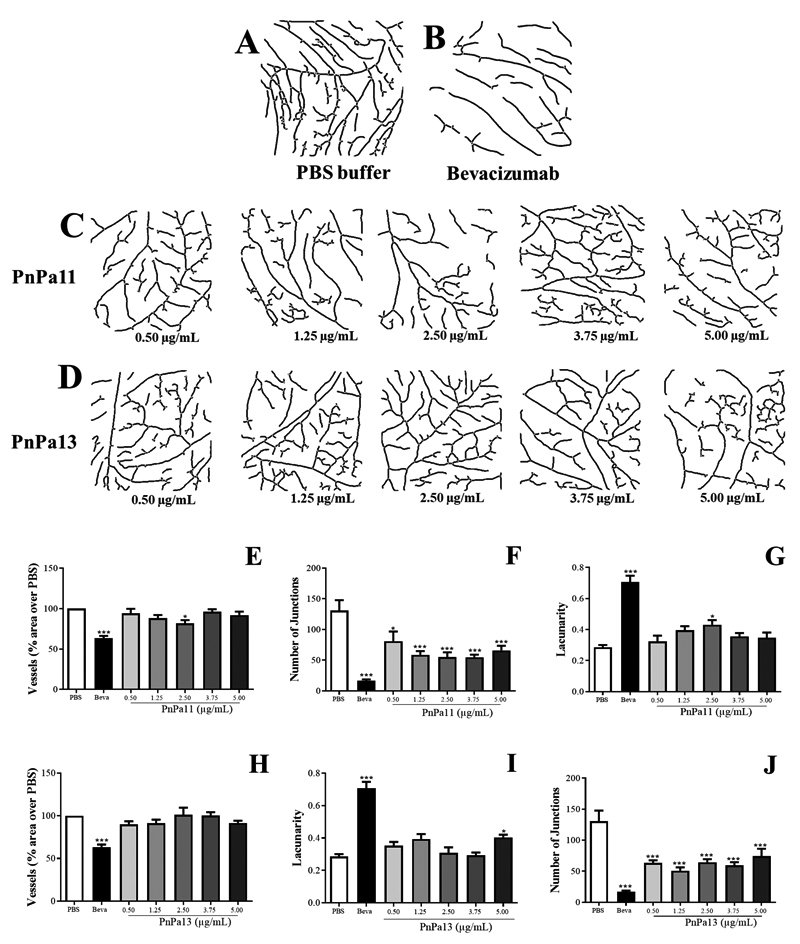
Microphotography of the vascular structure of CAM after injection by PnPa11 and PnPa13 peptides at different concentrations (0.50-5.00 µg/mL). **(A-D)** The photographs were obtained after processing procedure used to characterize the CAM vascular network. Graphs show the measure of vascularization after exposure to **(E-G)** PnPa11 or **(H-J)** PnPa13. Comparison among groups was analyzed using one-way ANOVA followed by Tukey post-test (n = 10). The data are represented by mean ± SD. The saline group was set as 100%. *Significant difference as compared to saline-group (*p < 0.05, ***p < 0.001).

The activity of the peptides in vessels was measured by CAM assays (Fig. 2A-2D). Bevacizumab treated group showed a reduction of 30% in vessels (Fig. 2E and 2H), an increase of lacunarity (150%) (Fig. 2F and 2I) and a decrease in the number of junctions (80%) with respect to the control group (Fig. 2G and 2J). On the other hand, the treatment with PnPa11 or PnPa13 did not induce a reduction in vessel area or an increase of lacunarity. However, a diminution in the number of junctions and, consequently, in the formation of new arterioles was observed (Fig. 2C, 2D, 2G, and 2J). 

### The intravitreal use of PnPa11 and PnPa13 does not compromise the visual function

To study the security of the intravitreal use of PnPa11 or PnPa13, ERG records taken 7 days after the intravitreal injection were evaluated. A difference in the pattern of ERG curves for the eyes treated with PnPa11 at concentrations above 1.25 µg/mL was observed (see Additional file 1). For PnPa13, no significant difference was verified. Also, statistical difference was detected for amplitudes of b- waves throughout the incidence of 0.01 cd·s·m^−2^ in eyes treated with PnPa11 at concentrations above 1.25 µg/mL (see Additional file 2).

### PnPa11 in high concentrations interferes in the intraocular pressure

PnPa13 did not induce any alterations in IOP. However, we did notice that PnPa11 caused a short reduction of pressure at 2.5 µg/mL (see Additional file 3A). Besides, PnPa13 did not cause hemorrhage during this process, or after 7 days of injection (see Additional file 3B). Furthermore, we did not observe significative alterations on the ONL thickness in the presence of the PnPa11 and PnPa13 at the maximum concentration tested (5.00 µg/mL) (see Additional file 4 that illustrates the histological images). Moreover, the ONL layer in the groups that received peptides was thicker than in the saline group, suggesting that an ongoing edema.

### PnPa11 and PnPa13 protect the retina from blue LED-induced degeneration

**Figure 3.**
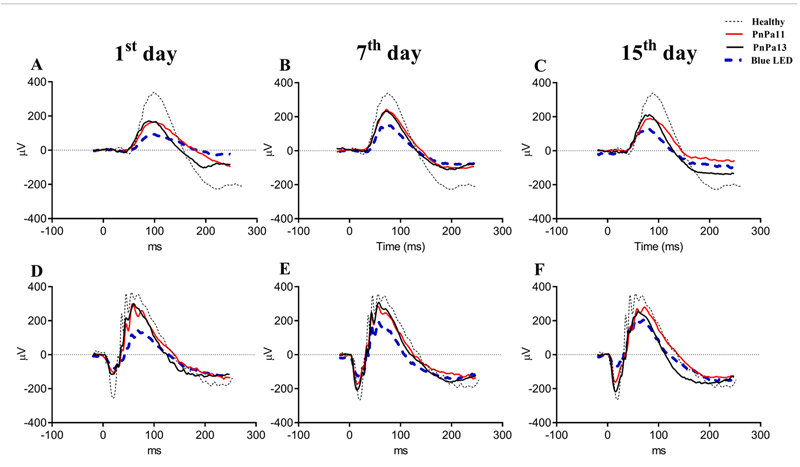
ERG curves at scotopic condition under luminous intensity of **(A-C)** 0.01cd·s·m^−2^ and **(D-F)** 3.0 cd·s·m^−2^. ERG responses were recorded **(A, D)** 1, **(B, E)** 7 and **(C, F)** 15 days after blue LED light exposure. ERG curves of eyes treated with saline, PnPa11 (1.25 µg/mL), and PnPa13 (1.25 µg/mL) were compared with the healthy group (n = 8). The pattern of ERG curves was analyzed by the Shapiro-Wilk test succeeded by Kruskal-Wallis and the post-test of Dunn.

For the investigation of the neuroprotective potential of PnPa11 and PnPa13, we evaluated the effects of PnPa11 and PnPa13 on the retinal degeneration process by electroretinography. Dark-adapted representative ERG records of 1, 7, and 15 days after the blue-LED light exposure were compared with non-exposed animals (healthy). Our ERG data demonstrated that light damage, induced by 2000 lux light, significantly reduced dark-ERG response amplitudes for all groups, after 24 hours (Fig. 3A-3D). However, this reduction was more severe in the saline-treated group, suggesting that synthetic peptides protected the retina against light-induced degeneration. On the 7^th^ (Fig. 3B-3E) and 15^th^ (Fig. 3C-3F) days after the blue LED light exposure, the ERG curves pattern of the blue-LED group showed a small retinal recovery. Meanwhile, the ERG curves patterns of peptides-treated groups were similar to those from the healthy groups, supporting that some electrophysiological activities of photoreceptors were preserved.

**Figure 4.**
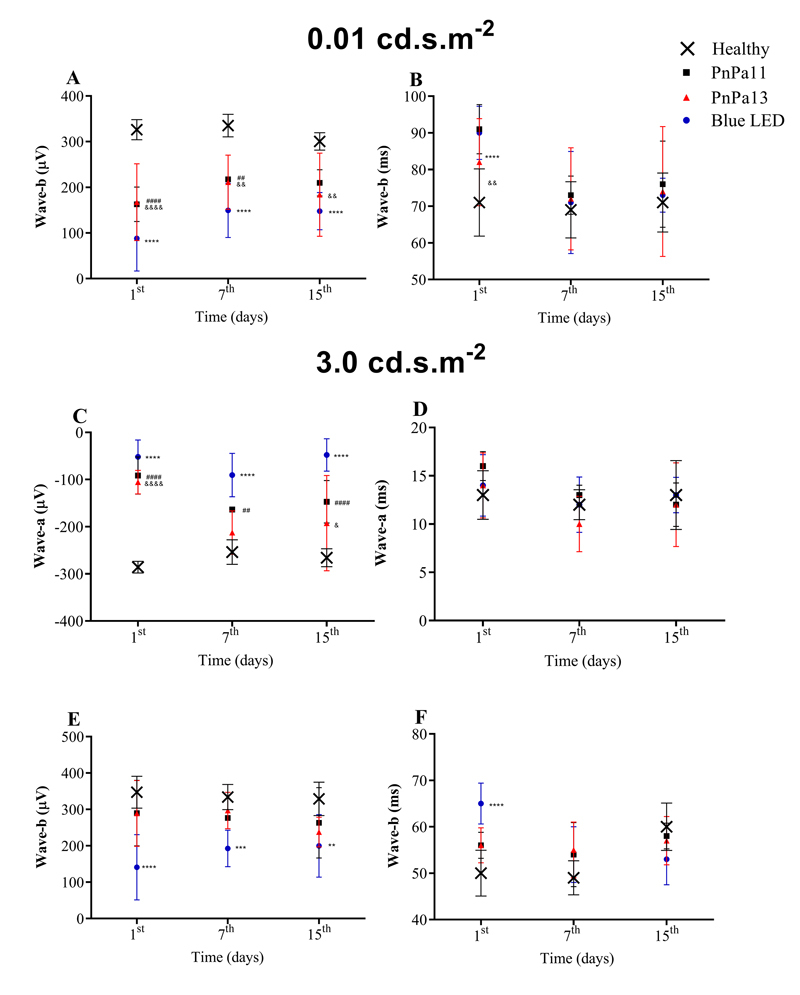
Media ± SD of a- and b- wave amplitude and implicit time at scotopic condition. **(A)** b-wave amplitude at luminous intensity of 0.01 cd·s·m^−2^. **(B)** b-wave implicit time at luminous intensity of 0.01cd·s·m^−2^. **(C)** a-wave amplitude at luminous intensity of 3.0 cd·s·m^−2^. **(D)** a-wave implicit time at luminous intensity of 3.0 cd·s·m^−2^. **€** b-wave amplitude at luminous intensity of 3.0 cd·s·m^−2^. **(F)** b-wave implicit time at luminous intensity of 3.0 cd·s·m^−2^. Data represent the means ± SD (n = 8). Comparison among groups was calculated using two-way ANOVA followed by Bonferroni post-test. *Significant differences as compared blue LED with healthy (**p < 0.01, ***p < 0.001, ****p < 0.0001). ^#^Significant differences as compared PnPa11 with healthy (^##^p < 0.01, ^####^p < 0.0001). ^&^Significant differences as compared PnPa13 with healthy (^&^p < 0.05, ^&&^p < 0.01, ^&&&&^p < 0.0001).

A significant decrease at the amplitudes of the b-(0.01 cd·s·m^−2^, Fig. 4A) and a-(3.0 cd·s·m^−2^, Fig. 4C) waves for all groups was observed after the blue LED light exposure. However, for b- waves (3.0 cd·s·m^−2^, Fig. 4E) only the saline treated-group displayed a significative reduction. In addition, PnPa11 and PnPa13 were able to inhibit changes at the implicit time of the b- wave (3.0 cd·s·m^−2^, Fig. 4F). These findings highlighted that the presence of PnPa11 and/or PnPa13 in the retina prevented the retinal dysfunction.

**Figure 5.**
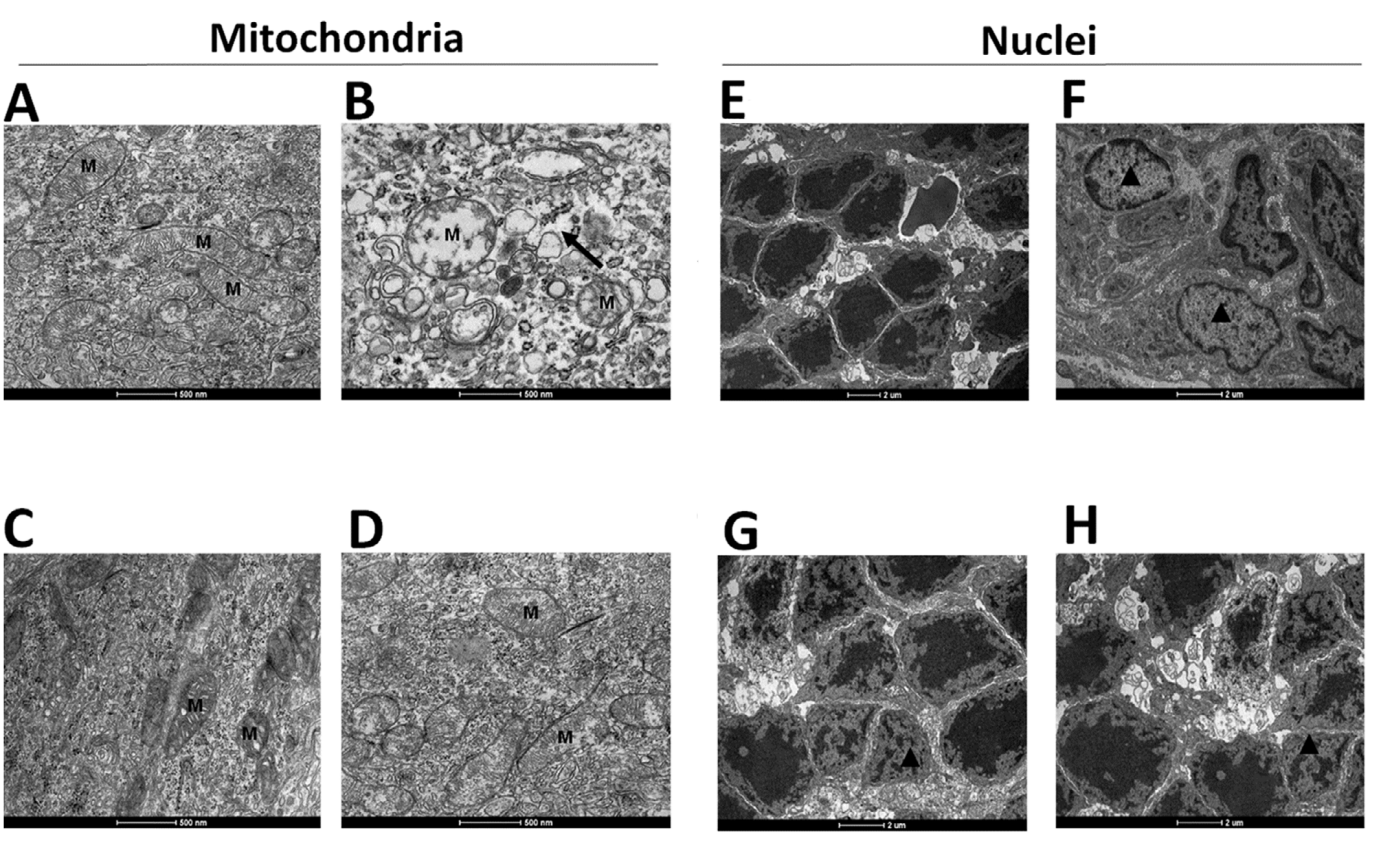
The ultrastructure of the retina after retinal degeneration blue LED-induced. A representative electron micrograph was taken from a healthy retina. The micrograph shows **(A)** normal appearance of the mitochondria (M) in comparison to **(B)** micrograph taken from mitochondria after blue LED light exposure that showed cristae fractured and vanished (M) and vacuolar degeneration (black arrow). In the retina that received **(C)** PnPa11 (1.25 µg/mL) or **(D)** PnPa13 (1.25 µg/mL) before light exposure, the mitochondria (M) showed a micrograph more preserved. In **€** note the photoreceptor nuclei in the ONL healthy retina, while in **(F)** nucleus pyknosis (▲) are seen after blue LED light exposure. Photoreceptors nuclei in the ONL show relatively normal nuclear appearance in the retina that received **(G)** PnPa11 (1.25 µg/mL) or **(H)** PnPa13 (1.25 µg/mL) prior to light exposure.

We examined the ultrastructural images of all groups 72 hours after the blue LED light exposure. The ultrastructure images of the healthy group showed healthy mitochondria (M) structures (Fig. 5A) and round and clear photoreceptor nuclei in the ONL (Fig. 5E). The blue-LED light exposure caused major injury in the photoreceptor layer. In saline-treated eyes, the mitochondria has wollen appearance, there was vacuolar degeneration (black arrow), and the cristae was fractured and vanished (Fig. 5B). Besides, nucleus pyknosis was observed (▲, Fig. 5F). In contrast, in the micrographs of the eyes that received PnPa11 or PnPa13 before light exposure, the mitochondria were more preserved (Fig. 5C and 5D) and the nuclei photoreceptors were partially protected (Fig. 5G and 5H).

**Figure 6.**
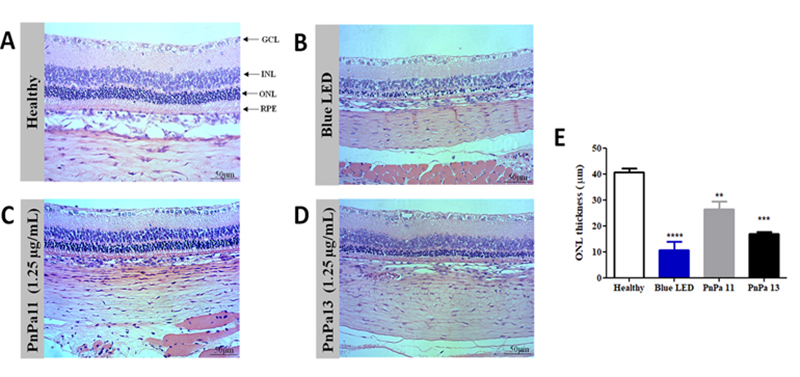
Histological retinal sections analysis. **(A)** Normal retinal layers in the healthy group compared to **(B)** blue LED light exposure-induced retinal injuries and treated eyes with **(C)** PnPA11 and **(D)** PnPA13. The ONL thickness retina of healthy group was compared to the blue LED light exposure group or treated with PnPa11 (1.25 µg/mL) or PnPa13 (1.25 µg/mL) before blue LED light exposure using one-way ANOVA followed by Tukey post-test. Three measures per slide were performed (n = 3). *Significant difference when compared to healthy group (**p < 0.01, ***p < 0.001, ****p < 0.0001). GCL: ganglion cell layer; INL: inner nuclear layer; ONL: outer nuclear layer; RPE: retinal pigment epithelium. Digital images were obtained with a 20× objective.

The analysis of histological sections from the eyes of the animals showed structural alterations in the retina (Fig. 6A-6D). A significant reduction in the thickness of ONL was observed in all groups exposed to the blue LED when compared to the healthy group. The average thickness of the ONL of the retinas varied between the samples (n = 3), being 40.74 ± 5.14 µm (healthy), 7.50 ± 3.76 µm (blue LED), 26.37 ± 9.50 µm (PnPa11) and 17.01 ± 2.26 µm (PnPa13). The decrease of the ONL layer was lowest in the groups treated with the peptides (Fig. 6E).

**Figure 7.**
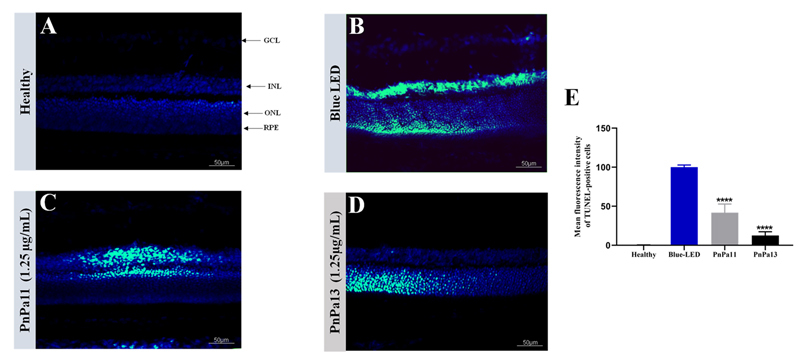
Evaluation of apoptotic cell death in blue LED retinal degeneration by TUNEL staining. Confocal micrographs were taken from vertical of sections of **(A)** healthy retina, **(B)** 2000-lux blue LED-exposed retinas, **(C)** PnPa11 (1.25 µg/mL) before blue LED light exposure and, **(D)** PnPa13 (1.25 µg/mL) before blue LED light exposure for **(E)** TUNEL staining are shown. Retinal layers were stained with DAPI (in blue) and TUNEL-positive cells are represented as green spots. Blue LED was set as 100% and mean **±** SD treated with PnPa11 (1.25 µg/mL) or PnPa13 (1.25 µg/mL) before blue LED light exposure using one-way ANOVA followed by Tukey post-test. Three measures per slide were performed (n = 3). *Significant difference as compared to healthy group (****p < 0.0001). Scale bar = 50 μm

By the TUNEL assay, no nuclei showed TUNEL-positive cells in the retinal layers from the healthy group (Fig. 7A). On the other hand, we found a strong positive staining in the INL and ONL layers in retinal sections from the blue LED-exposed group (Fig. 7B). In the eyes that received previous treatment with PnPa11 and PnPa13, a reduction of 58.24 ± 7.27 % and 87.54 ± 2.21% respectively was observed. Furthermore, in the PnPa11 group, TUNEL-positive cells were present in the INL and ONL layers (Fig. 7C). Whereas, for the PnPa13 group, the TUNEL-positive cells were present only in the ONL layer. 

**Figure 8.**
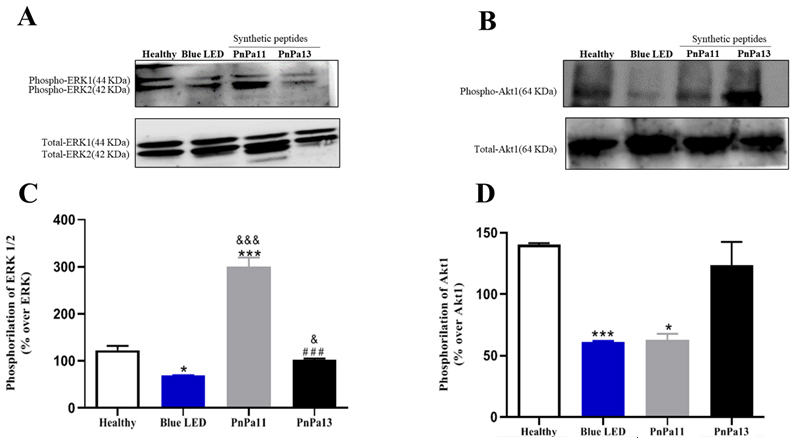
PnPa11 increases Erk1/2, but not Akt1 activation, while PnPa13 does not modulate Erk1/2 and Akt1 phosphorylation against Blue LED damage. Shown are representative immunoblots for **(A)** hosphor-(upper panel) and total-Erk1/2, **(B)** hosphor-(upper panel) and total-Akt1expression (lower panel) in retina of healthy rats, rats that were blue LED exposed (vehicle-treated), treated with PnPa11 (1.25 µg/mL), or PnPa13 (1.25 µg/m) before blue LED light exposure. About 100 mg of cell lysate was used for each sample. Graphs show the densitometric analysis of **(C)** hosphor-Erk1/2 or **(D)** hosphor-Akt1 normalized to total-Erk1/2 or total-AKT expression in retina of healthy rats and rats that were blue LED-exposed (vehicle-treated), treated with PnPa11 (1.25 µg/mL) or PnPa13 (1.25 µg/mL) before LED light exposure. Data represent the means ± SD of four independent experiments (healthy and PnPa11) and three independent experiments (blue LED and PnPa13), expressed as percentage of basal Erk1/2 or Akt1 phosphorylation. *Significant differences as compared with healthy (*p < 0.05). ^&^Significant differences as compared with blue LED (^&^p < 0.05). ^#^Significant differences as compared PnPa11 with PnPa13 (^#^p < 0.05). Analysis was carried out using two-way ANOVA followed by Bonferroni post-test.

We investigated the role of PnPa11 and PnPa13 on Erk1/2 and Akt1 activation. Blue LED caused a decrease of 54.42 ± 1.35% on p-Erk1/2 and of 78.76 ± 0.73% on p-Akt1 levels, compared to the healthy (Fig. 8A-8D). PnPa11 promotes an increase of 231.68 ± 19.62% on Erk1/2 phosphorylation compared to blue LED (Fig. 8A and 8C) but was not able to prevent Akt1 dephosphorylation (77.17 ± 4.90% reduction compared to healthy, Fig. 8B and 8D). By contrast, PnPa13 seems to be capable of preventing the p-Erk1/2 (an increase of 33.66 ± 3.25%) and p-Akt1 (an increase of 62.04 ± 19.14%) compared to blue LED. 

**Figure 9.**
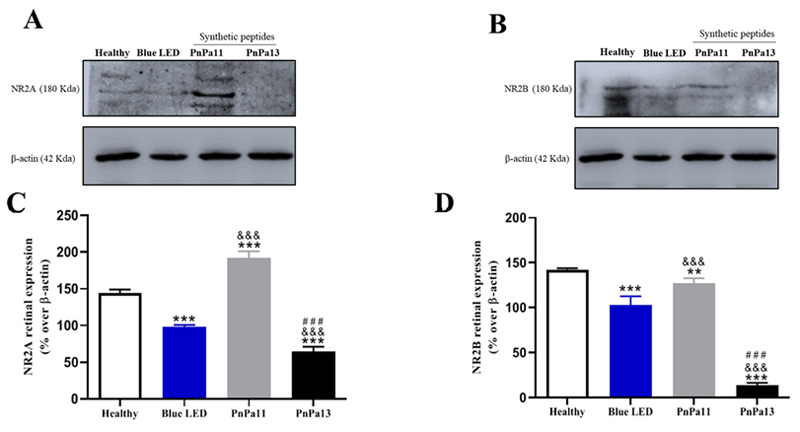
PnPa11 increases retinal expression NR2A and NR2B while PnPa13 reduces this expression in blue LED retinal stress model. Shown are representative immunoblots for **(A)** NR2A and β-actin expression (upper panel). **(B)** NR2B and β-actin expression (upper panel). Both expressions levels were performed in retina of healthy group, blue LED, or treated with PnPa11 (1.25 µg/mL) or PnPa13 (1.25 µg/mL) before LED light exposure. About 100 mg of cell lysate were used for each sample. Graphs show the densitometric analysis of NR2A or NR2B normalized to β-actin expression in retina of healthy rats and rats that were Blue LED exposed (vehicle-treated) or treated with PnPa11 (1.25 µg/mL) or PnPa13 (1.25 µg/mL) before LED light exposure. Data represent the means ± SD of four independent experiments (healthy and PnPa11) and three independent experiments (Blue LED and PnPa13), expressed as percentage of basal NR2A or NR2B phosphorylation. *Significant differences as compared with healthy group (*p < 0.05). ^&^Significant differences as compared with Blue LED (^&^p < 0.05). #Significant differences as compared PnPa11 with PnPa13 (^#^p < 0.05). Analysis was carried out using two-way ANOVA followed by Bonferroni post-test.

We also investigated the role of PnPa11 and PnPa13 on NMDA receptor subunits NR2A and NR2B expression. We found that blue LED caused a 45.29 ± 2.12% decrease on NR2A levels and 39.28 ± 9.55% decrease on NR2B levels with respect to healthy (Fig. 9A-9D). PnPa11 promoted an increase of expression of 93.07 ± 9.37% on NR2A and 23.96 ± 5.75% on NR2B compared to blue LED levels. On the other hand, PnPa13 promoted a significant drop in the levels of NR2A (34.22 ± 6.79%) and NR2B (89.44 ± 2.73%) expression in comparison to the ones exposed to blue LED.

## Discussion

ARPE-19 cells are present in many *in vitro* protocols that involve the investigation of ocular diseases [25,35]. In our study, we demonstrated that PnPa11 or PnPa13, within the range of tested concentration did not promote significant cytotoxic effects, indicating their safety for retina cells. These results consisted of an important step to support subsequent studies.

The CAM is a highly vascularized tissue of the avian embryo which mimics the biological vascular system of the human eye. Thereby, this method is very useful to investigate the antiangiogenic activity of new potential drugs [36]. Our results showed that PnPa11 and PnPa13 induced a clear reduction in the total number of junctions (vessels bifurcation). However, this shrinkage did not cause a significant decrease in vessel area in contrast to the saline group. Also, this reduction was less intense than that showed by the bevacizumab group. Bevacizumab is a monoclonal antibody widely used to inhibit vessel proliferation in retinal diseases [37].

Some studies have established mechanisms connecting retinal neurodegeneration processes with early microvascular irregularities that occur in eye diseases [38]. For example, diabetic retinopathy is characterized by the microcirculatory dysfunctions and angiogenesis that occurs due to chronic hyperglycemia. This effect is defined by the high loss of pericytes accompanied by the development of small vessels (capillaries) without blood perfusion [39]. Thus, the antiangiogenic effect is a potential target for eye disease treatment, and a large number of pharmaceutical research papers have highlighted this importance [40]. Therefore, our findings revealed that the reduction of the number of junctions by PnPa11 and PnPa13 could contribute to the retinal degeneration therapy. However, higher concentrations of these peptides should be tested for further investigation. 

For the first time, the intravitreal injections of PnPa11 and PnPa13 were investigated on the posterior segment of the eye. Although PnPa11 has demonstrated a sign of toxicity at doses higher than 1.25 µg/mL, PnPa13 did not present any evidence of toxicity within all tested concentrations. Since the b-waves are originated from the stimulus emitted by the electrical synapses between photoreceptors and bipolar cells, any change in this parameter may be related to an inner retinal impairment, especially, the bipolar cells or their connections with rods [41]. In this sense, PnPa13 demonstrated to be safe for intravitreal administrations in concentrations up to 5.0 µg/mL, and PnPa11 was safe for doses up to 1.25 µg/mL.

The IOP measures showed no change in intraocular pressure since the alteration observed in IOP was lower than 20% without clinical significance [42]. Also, there was no alteration in the eye fundus. In our study, the intravitreal procedure, as well as the injection of PnPa11 and PnPa13, did not compromise the retina function.

Earlier studies have shown that blue-LED light exposure is capable to cause damage to the photoreceptors [43]. These effects were more severe and longer lasting as exposure increases. Besides, as blue light is absorbed by the rhodopsin, a protein that converts light into an electrical signal and is localized in the photoreceptors, excessive blue light exposure can lead to apoptosis [44]. Thus, according to the ERG recordings in the retinal degeneration study (Fig. 3), we observed that the reduction in a- and b- waves amplitudes was higher in the vehicle-treated group. Also, the decrease of a-wave implicit time only took place in the vehicle-treated group (Fig. 4). All these findings confirmed that PnPa11 and PnPa13 were capable of preserving photoreceptors (rods and cones) when injected previously to the LED light exposure, and consequently, they could prevent retinal degeneration.

Histopathological analysis of TEM highlighted the damage to the mitochondria cristae in saline-treated eyes in the light stress model (Fig. 5). However, mitochondria showed a few modifications in those eyes treated with PnPa11 and PnPa13. These observations suggest that these peptides may reduce the death rate of light stress-induced photoreceptor cells, preserving these cells from mitochondrial injury [45,46]. 

Thickness measurements are often used to quantify light damage [47,48]. PnPa11 and PnPa13 were capable of respectively preventing approximately 65% and 42% of the ONL thickness reduction. Besides, the amount of TUNEL-positive cells in the ONL and INL was higher in the non-treated (blue-LED) group than in the healthy group. Moreover, a reduction of apoptotic cells was observed in the eyes treated with PnPa11 and PnPa13, which implies that these peptides could prevent apoptosis in retina. 

Both Akt and Erk1/2 signaling pathways are related to the antioxidative and antiapoptotic mechanisms [49]. Besides, both factors are involved with NMDA receptors signalling [50]. Furthermore, Yang et al. [51] have investigated a potential therapeutic approach for treating neurodegeneration in retina by evaluating Erk-1/2 and Akt signaling pathways.

The involvement of NMDA receptors in neuronal cell death in retina is well established. Some works have already demonstrated that different subunits of NMDA receptors trigger divergent pathways (proapoptotic and/or antiapoptotic) [52].

Choo et al. [52] showed that the activation of NR2B subunits provided a calcium influx into the mitochondria, which is a signal to the neuronal apoptosis. On the other hand, the activation of the NR2A subunit led to a pro-survival sign, which induces a neuron resistance to the glutamate insult. 

Our data demonstrated that the expression of the p-ERK1/2, p-Akt1, and NR2Aor NR2B subunits were altered by the blue LED light exposure. PnPa13 was able to inhibit the expression of NMDA receptors and avoid the dephosphorylation of Erk1/2. PnPa11 elicited the NR2A subunit, activating the neuronal pro-survival pathway, and avoiding the death of the neuronal cells. It also stimulated the phosphorylation of Erk1/2 and avoided the dephosphorylation of Akt. These findings provided a correlation between the neuroprotective effects of these peptides towards the neurodegeneration induced by blue-LED light exposure [49]. However, new studies are required for understanding the mechanisms that led to changes in the NMDA receptors expression, as well as to the phosphorylation of EKT1/2 and Akt.

## Conclusion

These synthetic peptides specifically act on oxidative and inflammatory stresses, often connected to neovascular complications and neurodegenerative diseases. Although the future challenges are numerous, it should be possible to understand how these peptides protect the retina against light-induced degeneration. Based on the present findings, it is possible to show the potential use of such compounds for retinal pathologies and their impact on life quality.

### Abbreviations

AMD: age-related macular degeneration; ARPE-19: Adult Retinal Pigment Epithelial cell line 19; ARVO: Association for Research in Vision and Ophthalmology; CAM: chicken chorioallantoic membrane; ERG: electroretinogram; HAc: acetic acid; INL: inner nuclear layer; IOP: intraocular pressure; ISCEV: International Society for Clinical Electrophysiology of Vision; LED: light-emitting diode; ms: milliseconds; NIH: National Institutes of Health; NMDA: N-methyl-D-aspartate; OCT: optimal cutting temperature; ONL: outer nuclear layer; PBS: phosphate buffer solution; ROS: reactive oxygen species; SRB: sulforhodamine B; TEM: transmission electron microscopy.

## Supplementary material

The following online material is available for this article:

Additional file 1. Representative ERG curves at scotopic condition 7 days after the intravitreal injection. ERG curves of eyes treated with different concentrations (0.5, 1.25; 2.50; 3.75 and 5.00 µg/mL) of PnPa11 and PnPa13 at luminous intensity of 0.01 cd·s·m^−2^ (A-C-E-G-I) and 3.0 cd·s·m^−2^ (B-D-F-H-J). All treated eyes were compared with received saline eyes (control) (n = 4). The pattern of ERG curves was analyzed by the Shapiro-Wilk test succeeded by Kruskal-Wallis and the post-test of Dunn.

Additional file 2. Media ± SD of a- and b-waves amplitude and implicit time at scotopic condition. **(A)** b-wave amplitude at luminous intensity of 0.01 cd·s·m^−2^. **(B)** b-wave implicit time at luminous intensity of 0.01 cd·s·m^−2^. **(C)** b-wave amplitude at luminous intensity of 3.0 cd·s·m^−2^. **(D)** b-wave implicit time at luminous intensity of 3.0 cd·s·m^−2^. **(E)** a-wave amplitude at luminous intensity of 3.0 cd·s·m^−2^. **(F)** a-wave implicit time at luminous intensity of 3.0 cd·s·m^−2^. The differences between amplitudes, implicit times of a-wave and b-wave were calculated using two-way ANOVA followed by Bonferroni post-test (n = 4). *Significantly different from saline group (*p < 0.05, **p < 0.01).

Additional file 3.
**(A)** Evaluation of IOP in eyes of rats. The pressure variation (mean ± SD) was calculated by the difference of the investigated eyes and the control group, in each measurement (n = 4). Statistical analysis was calculated using two-way ANOVA followed by Bonferroni post-test. *Significantly different from the control group (*p < 0.05). **(B)**Photograph of the fundus eye. The intravitreal injection of peptides does not affect retinal vessels. Black arrow indicates PnPP13 injected into the fundus eye.

Additional file 4. PnPa11 and PnPa13 do not alter the rat retinal morphology. Sequence of illustrative photographs of histological layers of the retina **(A)** saline, **(B)** PnPa11 (5.0 µg/mL), **(C)** PnPa13 (5.0 µg/mL) and **(D)** graph depicting the ONL thickness for each group. One-way ANOVA and Tukey’s test were performed for the statistical analysis (three measures per group, n = 3). A p-value < 0.05 was considered statistically significant. GCL: ganglion cell layer, INL: inner nuclear layer, ONL: outer nuclear layer, RPE: retinal pigment epithelium. Digital images were obtained with a 20× objective.
