# Supplementary material for: Intravitreal injection of peptides PnPa11 and PnPa13, derivatives of Phoneutria nigriventer spider venom, prevents retinal damage
Source: J Venom Anim Toxins Incl Trop Dis. 2020 Sep 23;26:e20200031. doi: 10.1590/1678-9199-JVATITD-2020-0031 (PMC7518191; doi:10.1590/1678-9199-JVATITD-2020-0031)

J Venom Anim Toxins incl Trop Dis

https://doi.org/10.1590/1678-9199-JVATITD-2020-0031

Supplementary material to “Intravitreal injection of peptides PnPa11 and PnPa13, derivatives of *Phoneutria nigriventer* spider venom, prevents retinal damage”

Additional file 1. Representative ERG curves at scotopic condition 7 days after the intravitreal injection. ERG curves of eyes treated with different concentrations (0.5, 1.25; 2.50; 3.75 and 5.00 µg/mL) of PnPa11 and PnPa13 at luminous intensity of 0.01 cd·s·m^{-2} (A-C-E-G-I) and 3.0 cd·s·m^{-2} (B-D-F-H-J). All treated eyes were compared with received saline eyes (control) (n = 4). The pattern of ERG curves was analyzed by the Shapiro-Wilk test succeeded by Kruskal-Wallis and the post-test of Dunn.

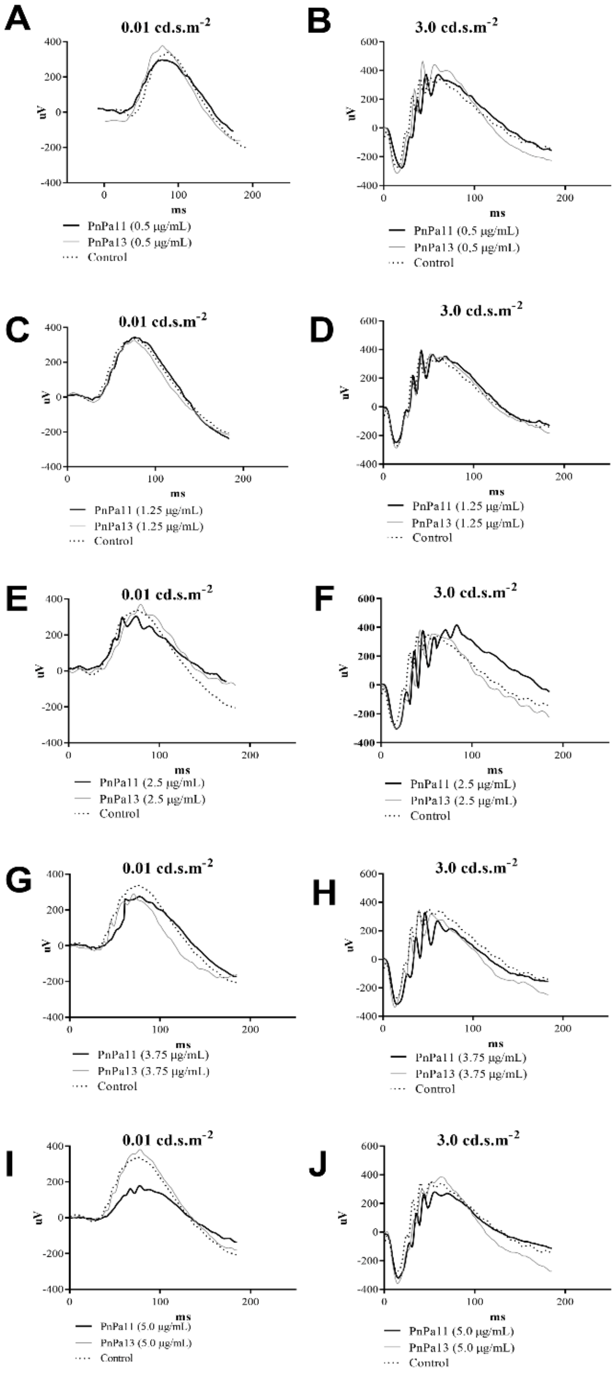

Supplement: Additional file 1. [file 1678-9199-jvatitd-26-e20200031-s1.pdf]
