# Supplementary material for: Intravitreal injection of peptides PnPa11 and PnPa13, derivatives of Phoneutria nigriventer spider venom, prevents retinal damage
Source: J Venom Anim Toxins Incl Trop Dis. 2020 Sep 23;26:e20200031. doi: 10.1590/1678-9199-JVATITD-2020-0031 (PMC7518191; doi:10.1590/1678-9199-JVATITD-2020-0031)

J Venom Anim Toxins incl Trop Dis

https://doi.org/10.1590/1678-9199-JVATITD-2020-0031

Supplementary material to "Intravitreal injection of peptides PnPa11 and PnPa13, derivatives of *Phoneutria nigriventer* spider venom, prevents retinal damage"

Additional file 2. Media ± SD of a- and b-waves amplitude and implicit time at scotopic condition. **(A)** b-wave amplitude at luminous intensity of 0.01 cd·s·m^{-2}. **(B)** b-wave implicit time at luminous intensity of 0.01 cd·s·m^{-2}. **(C)** b-wave amplitude at luminous intensity of 3.0 cd·s·m^{-2}. **(D)** b-wave implicit time at luminous intensity of 3.0 cd·s·m^{-2}. **(E)** a-wave amplitude at luminous intensity of 3.0 cd·s·m^{-2}. **(F)** a-wave implicit time at luminous intensity of 3.0 cd·s·m^{-2}. The differences between amplitudes, implicit times of a-wave and b-wave were calculated using two-way ANOVA followed by Bonferroni post-test (n = 4). *Significantly different from saline group (*$p < 0.05$, **$p < 0.01$).

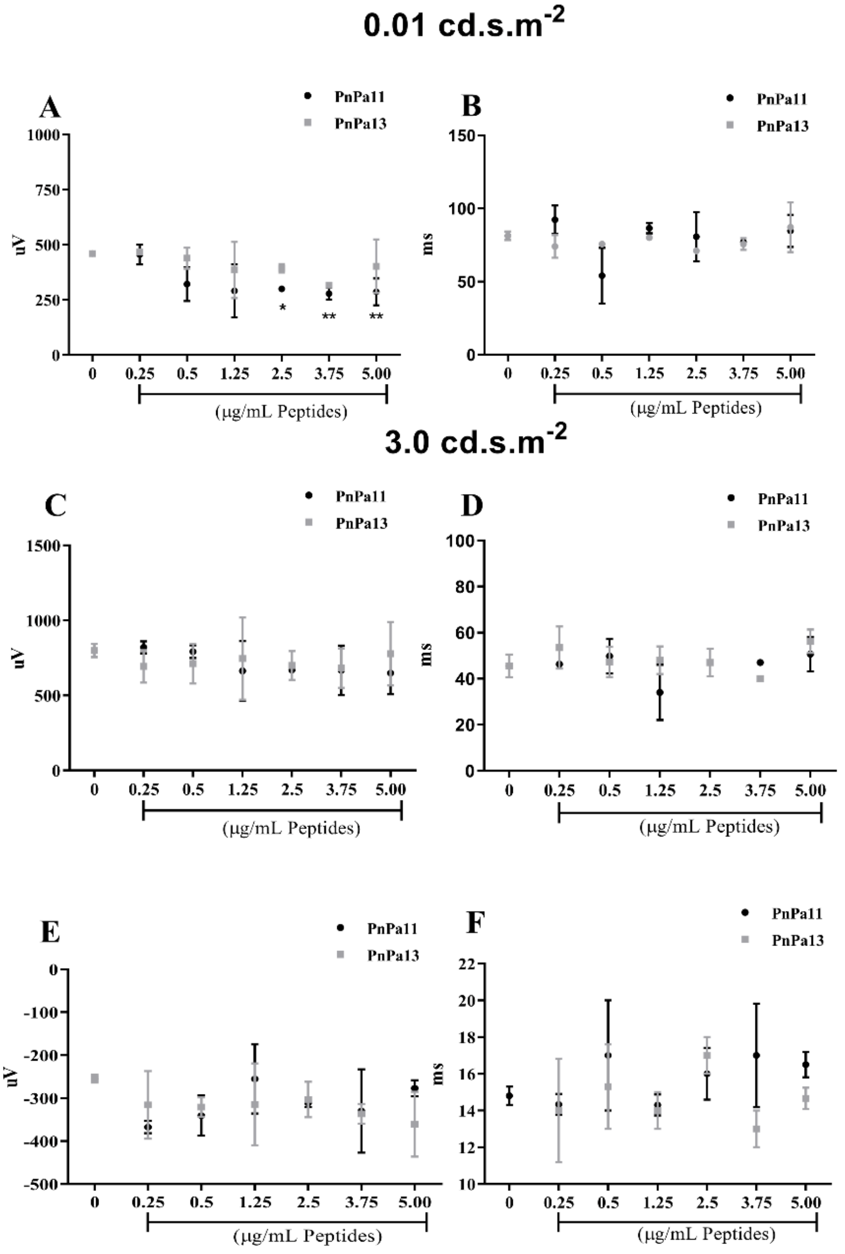

Supplement: Additional file 2. [file 1678-9199-jvatitd-26-e20200031-s2.pdf]
