# Supplementary material for: Intravitreal injection of peptides PnPa11 and PnPa13, derivatives of Phoneutria nigriventer spider venom, prevents retinal damage
Source: J Venom Anim Toxins Incl Trop Dis. 2020 Sep 23;26:e20200031. doi: 10.1590/1678-9199-JVATITD-2020-0031 (PMC7518191; doi:10.1590/1678-9199-JVATITD-2020-0031)

J Venom Anim Toxins incl Trop Dis

https://doi.org/10.1590/1678-9199-JVATITD-2020-0031

Supplementary material to "Intravitreal injection of peptides PnPa11 and PnPa13, derivatives of *Phoneutria nigriventer* spider venom, prevents retinal damage"

Additional file 3. (A) Evaluation of IOP in eyes of rats. The pressure variation (mean ± SD) was calculated by the difference of the investigated eyes and the control group, in each measurement (n = 4). Statistical analysis was calculated using two-way ANOVA followed by Bonferroni post-test. *Significantly different from the control group (*$p < 0.05$). **(B)** Photograph of the fundus eye. The intravitreal injection of peptides does not affect retinal vessels. Black arrow indicates PnPP13 injected into the fundus eye.

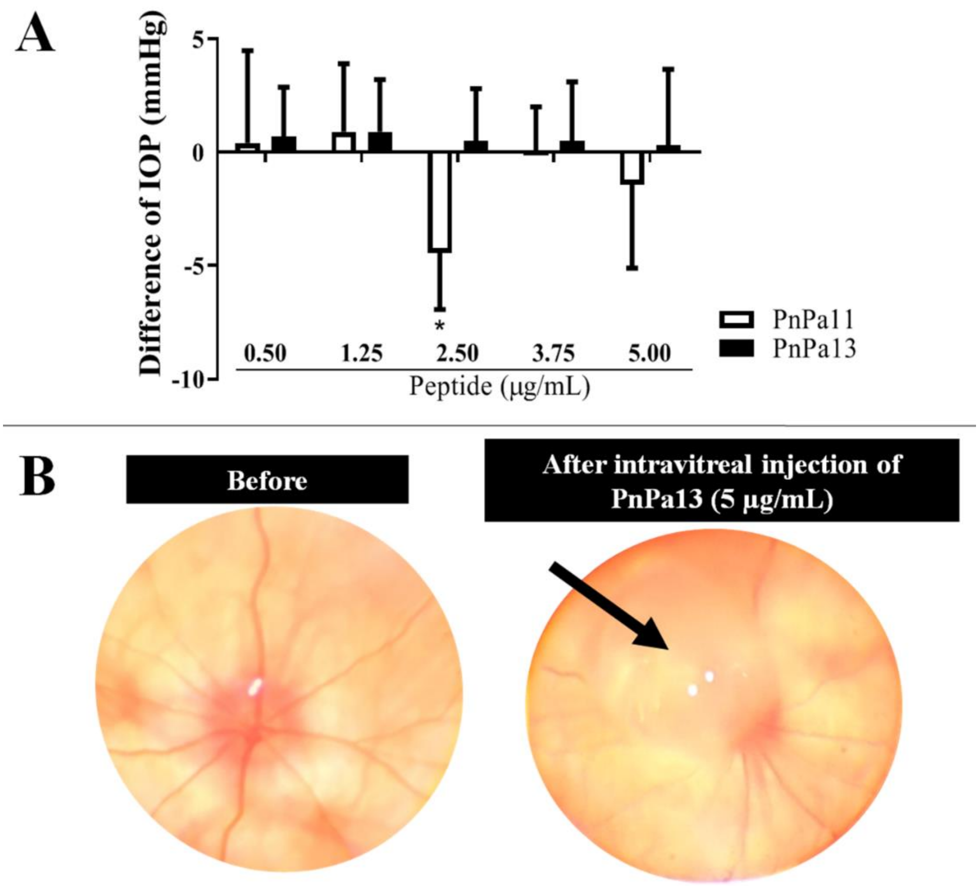

Supplement: Additional file 3. [file 1678-9199-jvatitd-26-e20200031-s3.pdf]
