# Supplementary material for: Intravitreal injection of peptides PnPa11 and PnPa13, derivatives of Phoneutria nigriventer spider venom, prevents retinal damage
Source: J Venom Anim Toxins Incl Trop Dis. 2020 Sep 23;26:e20200031. doi: 10.1590/1678-9199-JVATITD-2020-0031 (PMC7518191; doi:10.1590/1678-9199-JVATITD-2020-0031)

J Venom Anim Toxins incl Trop Dis

https://doi.org/10.1590/1678-9199-JVATITD-2020-0031

Supplementary material to "Intravitreal injection of peptides PnPa11 and PnPa13, derivatives of *Phoneutria nigriventer* spider venom, prevents retinal damage"

Additional file 4. PnPa11 and PnPa13 do not alter the rat retinal morphology. Sequence of illustrative photographs of histological layers of the retina **(A)** saline, **(B)** PnPa11 (5.0 µg/mL), **(C)** PnPa13 (5.0 µg/mL) and **(D)** graph depicting the ONL thickness for each group. One-way ANOVA and Tukey's test were performed for the statistical analysis (three measures per group, n = 3). A p-value < 0.05 was considered statistically significant. GCL: ganglion cell layer, INL: inner nuclear layer, ONL: outer nuclear layer, RPE: retinal pigment epithelium. Digital images were obtained with a 20× objective.

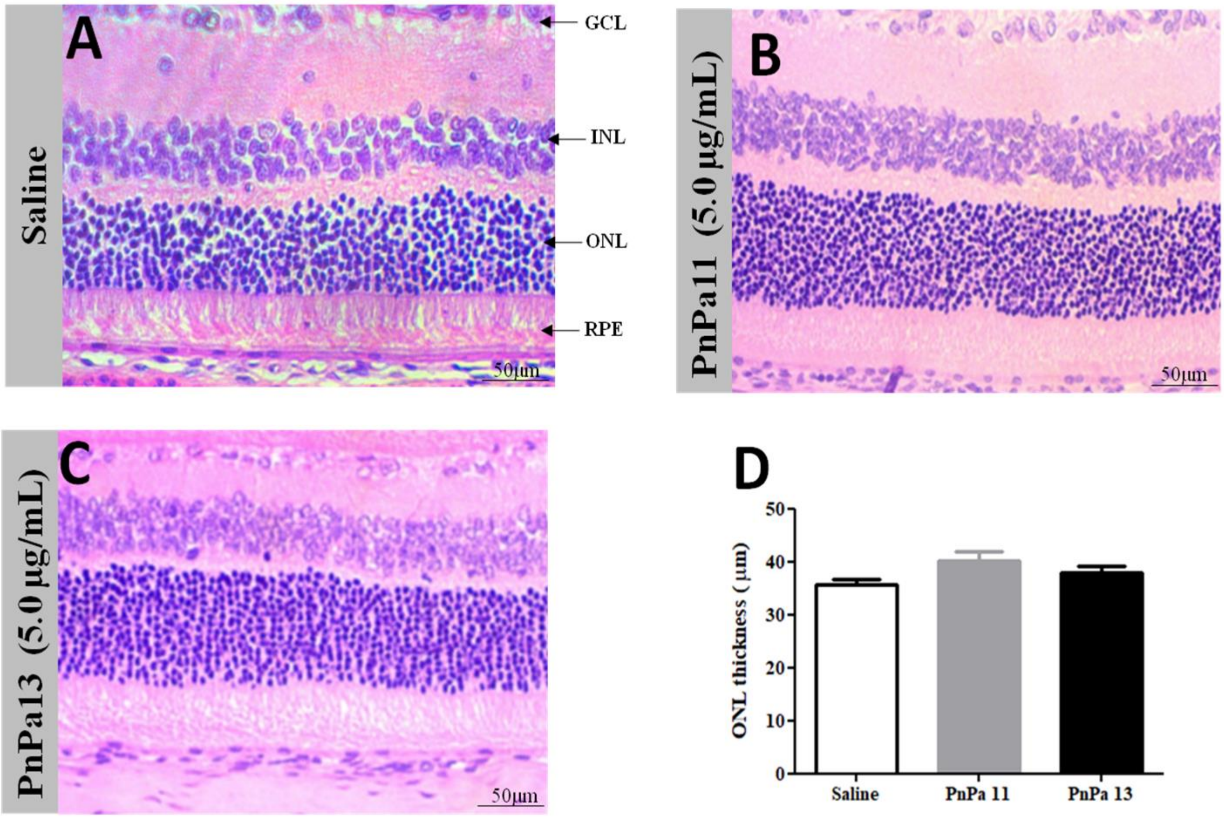

Supplement: Additional file 4. [file 1678-9199-jvatitd-26-e20200031-s4.pdf]
